# Supplementary material for: Exogenic origin for the volatiles sampled by the Lunar CRater Observation and Sensing Satellite impact
Source: Nat Commun. 2022 Feb 8;13:642. doi: 10.1038/s41467-022-28289-6 (PMC8825836; doi:10.1038/s41467-022-28289-6)
Supplement: Supplementary file 1 — Supplementary Information [file 41467_2022_28289_MOESM1_ESM.pdf]

Supplementary Information for

An Exogenic Origin for the Volatiles Sampled by the LCROSS Impact

K.E. Mandt[1*], O. Mousis[2], D. Hurley[1], A. Bouquet[2,3], K. Retherford[4,5], L. O. Magaña[4,5], and A. Luspay-Kuti[1].

[*]Corresponding author. Email Kathleen.Mandt@jhuapl.edu

The PDF file includes

Supplementary Methods
Reanalysis of LAMP Observations 2
Results 3
Volatile Sources 4
Model Description 8

Supplementary Tables
Table S1: Molecular abundances of LCROSS plume 6
Table S2: Elemental abundances of LCROSS plume and sources 7

Supplementary References 9

SUPPLEMENTAL MATERIAL

METHODS

Reanalysis of LAMP Observations

The LCROSS (*1*) impactor penetrated the lunar surface to a depth of 1-3 m (*2*). The plume was observed 30-60 seconds after impact by the Lunar Reconnaissance Orbiter (LRO) Lyman Alpha Mapping Project (LAMP), which detected H_2 and CO (*3*, *4*), and for the first 4 minutes after impact by the LCROSS spacecraft which measured the abundance of several additional species relative to water (*1*). The published abundances from LAMP did not include the observations that were made at the same time or covering the same region of space as the LCROSS measurements. To allow for direct comparison between the two sets of observations, we have reanalyzed the LAMP observations to find CO and H_2 abundances relative to water in the time period relevant to the LCROSS measurements.

The previous analysis of the LAMP data primarily focused on the pulse of vapor that expanded out in a spherical shell from the impact. This shell crossed LAMP's field of view in the period 30-60 s after impact, which was before LRO passed the impact site. LAMP's field of view was still > 50 km away from the impact site. A second feature was detected as LAMP's field of view passed above the impact site. This feature is more closely associated with the population of vapor detected by the LCROSS shepherding spacecraft. We used the model outlined in (*4*) to simulate that population of vapor for better comparison with abundances measured by the LCROSS Shepherding spacecraft. With this reanalysis, we find the sublimation rate of CO needed in the model to reproduce LAMP's observed count rate when it encountered the impact site. Although the initial impact is

thought to have produced heating of ~1000 K (*1*, *3*), this very hot material left the area very rapidly and is not included in the portion of the plume modeled here. The model assumes a sublimating source of CO from lofted material with starting velocities consistent with a Maxwell-Boltzmann distribution at temperature of 200 K. We determine the model column density as LAMP would detect it looking across the impact site at an altitude spanning from 30-40 km above the surface. To reproduce the LAMP observations, a source rate of 0.1 kg/s was required.

The particle dynamics are such that they disperse from the plume center quickly. Thus, we must make an assumption about the time profile of the source rate to relate LAMP measurements of the second peak, at 90 s after impact, to the LCROSS observations continuing until 3 minutes after impact. We assume that the CO source rate is constant throughout the first 150 seconds, which likely leads to an upper limit. With these assumptions, then the LAMP observations are consistent with <15 kg of CO released out of the ~5500 kg mass (*1*, *5*) of the LCROSS ejecta. Water mass in the regolith was estimated to be 5.6%, or ~300 kg, so CO is <5% relative to water observed by LCROSS.

Results

Table S1 provides the abundances of each species thought to be present in the plume or in the regolith based on the LCROSS observations using (*1*) for all species except for CO and H_2, which were recalculated as described above in the Reanalysis of LAMP Observations section. We provide two categories of abundances that depend on the type of ice present in the regolith. If we assume that the species measured in the plume were

released all at once by the destabilization of clathrates in the regolith, then the plume composition would represent the regolith composition. This category is designated as "clathrates". If we assume that all volatiles are condensed onto the surface individually, then their abundance in the plume is related to their volatility temperature. In this case we must estimate the abundance of the volatile in the regolith based on the total volume of regolith from which the volatile was released. The heating of the surface by the impact will result in temperatures that are highest at the impact point and decrease with distance from the impact. Volatiles with a lower volatility temperature will be released from a larger volume of regolith than volatiles that have a higher volatility temperature. We determined the abundance of each volatile in the regolith using the study conducted by (*6*) but correcting for the abundances of CO and H_2 that were calculated as part of this work. This category is designated as "condensates".

Volatile Sources

We determine the elemental composition for potential volatile sources based on reports of the source composition in the literature. Comet ice composition varies significantly, so we provide two values. The first value is for the coma of 67P/C-G, which has the most detailed reports of composition throughout the comet orbit of any comet, thanks to the *Rosetta* mission (*7*). Average values based on coma observations of several other comets (*7*), and provides a more realistic source composition representative of the possible range of values and their potential extremes. We illustrate two types of chondrites for chondritic source composition: CI, and CM (*8, 9*). These are carbonaceous chondrites,

which have the highest volatile composition of the chondrite classes and are frequently used to represent volatile-rich asteroid and meteoroid composition (e.g., *6*). The relative abundances of CO, H_2O, H_2, OH, and S in volcanic gas was estimated by (*10*) based on a review of Apollo sample composition measurements. Nitrogen abundance in lunar volcanic gas was determined by (*11*) based on lunar sample composition, which suggests that N/C in lunar volcanic gas is an order of magnitude larger than terrestrial volcanic gas. We also reviewed lunar exosphere measurements to ensure that our source composition included potential volatiles provided by ongoing interior outgassing on the Moon through events described as Transient Lunar Phenomena (*12*). The lunar exosphere is primarily made up of helium, neon, and argon (*13*). Trace amounts of CH_4 and NH_3 have been detected, and are interpreted to be derived from solar wind carbon and nitrogen (*14,15*). Although the argon isotope, ^{40}Ar, comes from the interior of the Moon through radiogenic decay of heavier elements, no carbon-, nitrogen-, oxygen-, or sulfur-bearing species have been clearly connected to related internal diffusion and outgassing processes (*16*). Therefore, we assume that TLP composition is similar enough to volcanic gas composition determined by the studies outlined in (*10*,*11*). However, as noted by (*12*), studying the composition of TLPs is essential now in their pristine state, before extensive robotic and human exploration of the Moon introduces large amounts of anthropogenic gas to the exosphere and TLPs.

Table S1.

Molecular abundance of the volatiles observed in the LCROSS impact plume relative to water and their predicted abundance in the regolith if the volatiles are stored as condensed material. The abundance in the LCROSS plume was measured by the LCROSS experiment (*1*) and LRO-LAMP (*3*). The LAMP observations have been reanalyzed to obtain abundances measured at the same time as the LCROSS abundances (*This work*). The volatiles measured in the plume are assumed to be the same composition as in the regolith if the volatiles are stored as clathrates. This is because all volatiles are released together when clathrates destabilize. The estimates for volatiles condensed on regolith grains are based on the analysis of each species volatility temperature conducted by (*6*) correcting CO and H_2 to agree with LAMP observations. Species in **bold** are used for the atmospheric escape analysis.

	LCROSS plume or "Clathrates"	Condensed on regolith (*6*) (%) or "Condensates"	Volatility temperature (K) (*17*)	Mass (amu)
**H_2O**	100±27.5 (*1*)	5.60±1.54	100.8	**18**
**OH**	0.030±0.001 (*1*)	n/a		**17**
**H_2**	6.0±5.9 (*This work*)	0.050±0.049 (*This work*)		**2**
CH_4	0.65±0.41 (*1*)	0.0030±0.0019	22.0 (*18*)	16
C_2H_4	3.12±2.46 (*1*)	0.020±0.016	40.0	28
**CO**	2.00±1.99 (*This work*)	0.0800±0.0796 (*This work*)	16.8	**28**
**CO_2**	2.17±1.38 (*1*)	0.040±0.025	53.4	**44**
CH_3OH	1.55±7.92 (*1*)	0.10±0.51	90.0	32
S	Not observed	Not observed	181 (*6*)	32
**H_2S**	16.75±2.82 (*1*)	0.200±0.034	47.8	**34**
**SO_2**	3.19±0.08 (*1*)	0.200±0.005	70.5	**64**
**OCS**	Not observed	Not observed	46.8 (*18*)	**60**
**NH_3**	6.03±1.26 (*1*)	0.070±0.034	63	**17**
**N_2**	Not observed	Not observed	16.2 (*18*)	**28**

Table S2 applies the species composition from Table S1 to calculate the elemental abundances for the regolith ices if they are “clathrates” or “condensates”. Note that the greatest difference between the elemental abundances for the two types of ice are found in the nitrogen and oxygen relative to carbon. We also include the elemental abundances used for the sources shown in Fig. 1 based on the references indicated in the main article text, Section 6.3, and in the table references. Uncertainties are propagated from measured values reported in the literature to the elemental ratio uncertainties by standard means for error propagation. These elemental ratios and their uncertainties were used in the model described below for each of the cases outlined in Table 1 of the main text.

Table S2.

Elemental abundance of the volatiles in the top 1-3 meters of regolith in the Cabeus Crater PSR sampled by the LCROSS impact compared to possible sources for the volatiles. The LCROSS plume abundance assumes that the volatiles are stored as clathrates. The regolith abundance assumes that the volatiles are stored as condensates and that each species is released according to its volatility.

	LCROSS plume (*1*) or “Clathrates”	Regolith (*6*) or “Condensates”	67P/C-G (*7*)	Average comets (*7*)	CI Chondrites (*8, 9*)	CM Chondrites (*8*)	Volcanoes (*10, 11*)
**C/S**	0.63±0.36	0.66±0.38	5.35±0.16	≥ 2.16	0.68±0.02	0.59±0.02	0.92±0.47
**N/C**	0.48±0.24	0.27±0.14	0.10±0.05	≤ 0.37	0.085±0.003	n/a	0.006±0.001
**O/C**	9.37±4.36	23.7±11.0	12.06±4.06	50.0±49.7	12.58±0.38	20.80± 0.62	1.02±0.01
**C/H**	0.04±0.03	0.02±0.01	0.05±0.02	0.01± 0.0099	2.35±0.07	1.69±0.05	17.04±5.14

Model Description

We modeled the possible mixtures of sources by calculating C/S, N/C, O/C, and C/H for every combination of percent contribution of volcanic, chondrite, cometary, and solar wind contributions that add up to 100%. All elemental ratios used as input to the model are shown in Table S2 with the exception of solar wind contribution. Each time that we applied the model, we tested 167,002 possible combinations of these sources. In all cases, the solar wind was assumed to contribute only hydrogen and oxygen to the ratios by delivering water to the PSR. This is because the solar wind nitrogen and sulfur are orders of magnitude lower than solar wind hydrogen and any contribution would be negligible compared to other potential volatile sources as seen in the right panel of Fig. 1. The other sources would deliver all four elements according to their abundances in the source.

The model was applied five times using the constraints outlined in Table 1. We did not apply the model for a volcanic atmosphere because we were only evaluating one source in this case. A volcanic source was allowed in all five cases and no models with volcanic contributions could fit in any of these cases because of the deficiency in nitrogen in volcanic gas. When fitting within the uncertainties, a modeled mixture of sources was determined to be an accurate fit if the simulated elemental ratios and uncertainties overlapped with the observations by LCROSS and the uncertainties in the measurements.

Supplementary References

1. A. Colaprete et al., Detection of water in the LCROSS ejecta plume. *Science,* **330**, 463–468 (2010). doi:10.1126/science.1186986
2. P. O. Hayne, B. T. Greenhagen, M. C. Foote, M. A. Siegler, A. R. Vasavada, D. A. Paige, Diviner lunar radiometer observations of the LCROSS impact. *Science*, **330**, 477–479 (2010). doi:10.1126/science.1197135
3. G. R. Gladstone et al., LRO-LAMP observations of the LCROSS impact plume. *Science* **330**, 472–476 (2010). doi:10.1126/science.1186474
4. D. M., Hurley et al., Modeling of the vapor release from the LCROSS impact: 2. Observations from LAMP. *Journal of Geophysical Research: Planets,* **117**, (2012). doi:10.1029/2011JE003841
5. Schultz, P. H., Hermalyn, B., Colaprete, A., Ennico, K., Shirley, M., & Marshall, W. S. The LCROSS cratering experiment. *Science*, **330**, 468-472 (2010). doi:10.1126/science.1187454
6. A. A. Berezhnoy, E. A. Kozlova, M. P. Sinitsyn, A. A. Shangaraev, V. V. Shevchenko. Origin and stability of lunar polar volatiles. *Advances in space research,* **50**, 1638–1646 (2012). doi:10.1016/j.asr.2012.03.019
7. M. Rubin et al., Elemental and molecular abundances in comet 67P/Churyumov-Gerasimenko. *Monthly Notices of the Royal Astronomical Society,* **489**, 594–607 (2019). doi:10.1093/mnras/stz2086
8. C. M. O.'D. Alexander, Quantitative models for the elemental and isotopic fractionations in chondrites: The carbonaceous chondrites. *Geochimica et Cosmochimica Acta*, **254**, 277-309 (2019). doi:10.1016/j.gca.2019.02.008
9. K. Lodders, Solar system abundances of the elements. In *Principles and perspectives in cosmochemistry* (pp. 379-417). Springer, Berlin, Heidelberg (2010). doi: 10.1007/978-3-642-10352-0_8
10. D. H. Needham, D. A. Kring, Lunar volcanism produced a transient atmosphere around the ancient Moon. *Earth and Planetary Science Letters,* **478,** 175–178 (2017). doi:10.1016/j.epsl.2017.09.002
11. B. Fegley, Thermodynamic models of the chemistry of lunar volcanic gases. *Geophysical Research Letters,* **18**, 2073–2076 (1991). doi:10.1029/91GL02624
12. Crotts, A. P. (2008). Lunar outgassing, transient phenomena, and the return to the Moon. I. Existing data. *The Astrophysical Journal*, **687**(1), 692. doi:10.1086/591634
13. J. H. Hoffman, & Hodges, R. R. (1975). Molecular gas species in the lunar atmosphere. *The Moon*, **14**(1), 159-167. doi:10.1007/BF00562981
14. R. R. Hodges Jr (2016). Methane in the lunar exosphere: Implications for solar wind carbon escape. Geophysical Research Letters, **43**(13), 6742-6748. doi:10.1002/2016GL068994
15. N. R. Mukherjee (1981). Solar-wind interactions with the moon: Nature and composition of nitrogen compounds. *The moon and the planets*, ***25***(4), 451-463. doi:10.1007/BF00919079

16. R. M. Killen (2002). Source and maintenance of the argon atmospheres of Mercury and the Moon. *Meteoritics & Planetary Science*, ***37***(9), 1223-1231. doi:10.1111/j.1945-5100.2002.tb00891.x
17. Fray, N., & Schmitt, B. (2009). Sublimation of ices of astrophysical interest: A bibliographic review. *Planetary and Space Science*, **57**(14-15), 2053-2080. doi:10.1016/j.pss.2009.09.011
18. J. A. Zhang, D. A. Paige, Cold-trapped organic compounds at the poles of the Moon and Mercury: Implications for origins. *Geophysical Research Letters,* **36** (2009). doi:10.1029/2009GL038614
